# “There's always something, and then there's more”: a qualitative examination of how virtual peer led self-management can create community around the evolving SCI experience

**DOI:** 10.3389/fresc.2024.1377218

**Published:** 2024-04-29

**Authors:** Amy J. Starosta, Shannon Tyman, Chris Garbaccio, Jayden Chapman, Jeanne M. Hoffman

**Affiliations:** Department of Rehabilitation Medicine, University of Washington School of Medicine, Seattle, WA, United States

**Keywords:** spinal cord injury, self-management intervention, peer mentoring, qualitative analysis, telemedicine

## Abstract

**Introduction:**

Self-management programs for spinal cord injury (SCI) are a growing adjunct to traditional healthcare services aiding individuals with SCI in learning and managing symptoms and medical care. A benefit of self-management programs is that they can be facilitated by peers, offering a unique lived experience of adjusting to and managing SCI. While a growing body of literature highlights the effectiveness of peer led programs, there is limited understanding of how individuals engage with peer programs or critical components of peer support. The current study seeks to understand how individuals engaged with peers in the context of a self-management program.

**Methods:**

Secondary qualitative analysis of online forum posts resultant from a peer led self-management course for SCI.

**Results:**

Content analysis revealed several themes of how participants engaged with members of the group, including skill building, resource sharing, and problem solving. A process level theme of emotional connection to others living with similar SCI-related challenges was defined as “bearing witness.” Participants commented frequently that groups were the first time they engaged with a community with lived experience, and shared experience was frequently highlighted in the responses from individuals as one of the most unique and important aspects of the intervention.

**Discussion:**

Themes identified suggest that bearing witness was a critical component of peer led intervention. While self-management content provided structure for engagement and discussion, participants report that connectedness and shared experience made content more impactful and relevant. Future research should examine if alignment of peer and participant experience increases the impact of interventions and explore if this theme is important for other chronic medical populations.

## Introduction

Spinal cord injury can cause multisystem changes in physical function as well as broad impacts on daily activities of living, occupational and relational changes, and impacts on mood. As individuals are adjusting to SCI, they benefit from physiatry, therapies (which can include physical, occupational, speech/language, recreational, and vocational), and rehabilitation psychology. While these professional services are all important components of ongoing medical management and rehabilitation, they may not be able to fully address the lived experience of adjusting to SCI. Because of the needs that exceed the expertise of the interdisciplinary team, there is an increasing emphasis on peer support and mentorship as an important (1) and understudied component of SCI care (2).

The World Health Organization now recommends the utilization of non-health professionals such as peers to assist in the delivery of a comprehensive range of health care and rehabilitation services (1). A recent scoping review found increased empirical evaluation of the impact of SCI peer-led interventions over the past 10 years (2). Face-to-face peer support programs have been found to improve self-efficacy and reduce the occurrence of medical complications (3), including hospital readmission (4). Additionally, peer support is positively associated with social participation and life satisfaction (5). These peer programs show significant promise but many of them continue to rely on a one-on-one format, often focusing on one peer to one patient. While a growing body of literature highlights the effectiveness of peer led programs, there is limited understanding of how individuals engage with peer programs or what constitutes a critical component of peer support. An initial review of the existing literature suggests that credibility, equitability, mutuality, and normalization are all key factors for an effective peer led intervention (6).

Health education or self-management programs are a growing adjunct to traditional health care services that aid individuals with chronic medical conditions in learning about and managing their symptoms and medical care. These programs have been effective with a wide range of chronic health and rehabilitation conditions, including for individuals living traumatic brain injury (7) or SCI (8). The emphasis on self-management rather than medical management provides a natural role for individuals that have lived experience and success in self-management offering peer-mentorship and support. Our team developed SCI Thrive, a peer-led online self-management program designed to connect individuals living with SCI to support from peers, SCI experts, and self-management training. SCI Thrive integrates cognitive behavioral self-management strategies with SCI specific examples created in partnership with our peer leaders and SCI experts. The self-paced intervention is paired with a peer-led group of 6–10 participants delivered via video conferencing. Additionally, participants engaged in an online forum to support communication throughout the 6-week group. The online format was especially beneficial during the era of COVID, however, the intent of the design was to focus on online delivery of the content regardless of social distancing recommendations. While initial quantitative analyses indicate that the intervention is effective in improving individual self-efficacy (7), feedback from participants and peer leaders repeatedly highlighted the importance of the group process as a main benefit of the intervention. This suggested that exploring only the quantitative outcomes may miss some of the richness of the intervention and the lived experience of the participants in the groups.

Therefore, the study team, including the peer leaders, conducted qualitative analyses of the written content in the online forum to explore the rich, unstructured interactions that occurred between participants. The current study seeks to understand how participants engaged with peers, both leaders and participants, in the context of a self-management program.

## Materials and methods

### Context

This qualitative study was part of a clinical trial designed to assess the efficacy of SCI Thrive. Full description of the methods for recruitment, retention, intervention, and assessment are described elsewhere (8). In short, all participants provided informed consent prior to initiation of any study procedure. Enrolled participants were grouped into concurrent cohorts. Each cohort had access to a self-paced online course, focused on teaching self-management skills that are known to be effective for successful health management utilizing written information about the skills with relevant examples. As participants engaged with the course content, they also attended 4 online group meetings facilitated by peer-mentors trained in the self-management intervention and group facilitation. These meetings were spaced over the 6-week intervention.

In addition to the group meetings, participants also interacted in online forums. Participants were directed to utilize the forum to discuss the materials in each module. In addition to responding to course content, participants were also encouraged to use the forums in whatever fashion they found to be helpful, such as to share resources with one another. While some conversation threads were initiated by peer leaders, participants also had the freedom to start their own topics and reply to other's posts. All posts were visible to all group members but specific and private to each cohorted group.

### Qualitative analysis

Analyses centered on understanding the usage of the online forum by participants and interactions between participants. Content review and data analyses were conducted using ATLAS.ti. Data analyzed included all online posts made by participants in the forum associated with the SCI Thrive intervention. All peer-leader posts were excluded from the current content analysis. The team's initial approach focused on directed content analysis (9) while subsequent analyses utilized a collaborative thematic analysis (10, 11). First steps included directed content analysis by identifying the appearance and frequency of keywords throughout the content (12). These keywords were based on the personal and group facilitation experience of SCI Peers as well as expert opinion. After initial directed content analysis was conducted by one author (ST), all authors reviewed frequency and context of key words (total of 5 coders). Group discussion focused on identifying the underlying context in which these words were used as well as the themes in the forum discussions. After group consensus was reached regarding the latent themes, operational definitions of the themes were created (see Table 1). Team members individually reviewed all forum content, coding for the presence of identified themes. Coded quotes were grouped and reviewed as a group, with hallmark quotes identified to best illustrate the identified theme.

**Table 1:** Themes and operational definitions.

Theme	Operational definition
Resource sharing	Sharing specific resources outside of SCI Thrive such as websites and names of equipment
Skill building	Practicing skills learning in SCI Thive Goal setting Mindfulness practices (guided imagery, breathing, autogenic relaxation) Tackle a problem & making decisions Thinking about thinking Getting what you need from others Taking charge of your thoughts Working on goals
Problem solving	Sharing possible solutions to problems unique to people with SCI
Bearing witness	Responding to or sharing experiences related to SCI that people without SCI may not understand. Expressions of care, encouragement, or empathy. Expressions of frustrations and success. Storytelling & responding to storytelling.

## Results

In total, 184 individuals with SCI participated in the study over 22 unique cohorted groups. Characteristics of the total sample was reviewed in previously published work (8). Of the 184 participants, 26 individuals did not create an online login for the course and never engaged with online content, and as such were removed from the current qualitative analyses. Table 2 reviews demographic information for the 158 participants who accessed the online content. Among the individuals who engaged with the course content, 84% (*n* = 133) created at least one post in the online forum. An additional 25 individuals logged in to the online course and forum but did not post in the forum. These individuals may have benefited from reading discussions within their cohort, without creating any content themselves. The range of engagement with the forum was wide: on average participants made 8.6 forum posts over the 6-week course, but engagement ranged from 1 post up to 58 posts by an individual. Cohorted groups varied in the amount of forum activity. On average, group forums had 51.7 posts. These posts tended to be split between first posts in a topic (*M* = 22.1) and replies to someone else's post (*M* = 29.6).

**Table 2:** Demographics.

Patient characteristics	
Age	
Mean (SD) years	51.2 (15.1)
Sex	*N* (%)
Male	89 (56%)
Female	69 (44%)
Race	
White	125 (79%)
Black	9 (6%)
Native American/Alaskan	3 (2%)
Asian/Pacific Islander	13 (8%)
Other	8 (5%)
Race/Ethnicity	
White Non-Hispanic	113 (72%)
Non-White Non-Hispanic.	29 (18%)
Hispanic	15 (10%)
Unknown	1
Marital status	
A. Never Married	46 (29%)
B. Married/Partnered	87 (55%)
C. Other	25 (16%)
Unknown	0
Education	
Mean (SD) years (*approx*.)	15.5 (2.5)
HS/GED or less	28 (18%)
Undergraduate degree	81 (52%)
Postgraduate degree	44 (28%)
Some Coll./Trade	4 (3%)
Unknown	1
Income	
<$25k	24 (18%)
$25k to <$50k	22 (16%)
$50k to <$75k	31 (23%)
$75k or more	59 (43%)
Unknown	22
Injury characteristics	
Years since injury	
Mean (SD, range)	15.6 (14.1, 0.1–54.7)
0–1	11 (7%)
>1–5	33 (21%)
>5–10	36 (23%)
>10–20	31 (20%)
>20	47 (30%)
Injury level	
(A) Cervical	94 (59%)
(B) Thoracic	51 (32%)
(C) Lumbar	13 (8%)
Injury type	
Incomplete	96 (63%)
Complete	56 (37%)
Unknown	6
ASIA	
A	24 (36%)
B	12 (18%)
C	11 (16%)
D	19 (28%)
E	1 (1%)
Unknown	91
Home and region descriptors	
Census region	
Midwest	18 (12%)
Northeast	19 (13%)
South	26 (17%)
West	89 (59%)
Unknown/NA	6
Urban-rural class	
Rural	5 (3%)
Micropolitan	6 (4%)
Metropolitan	142 (93%)
Unknown/NA	5
Living situation	
Private residence	154 (97%)
Other	4 (3%)
Unknown	

### Overview of themes

Content analysis revealed several themes, many of which were a direct reflection of the course content, such as Resource Sharing, Skill Building, or Problem Solving. Throughout the forum posts, there was a deeper element of empathy and shared experience that happened organically and permeated all themes. This process level theme of emotional connection to others living with similar SCI-related challenges was defined as “Bearing Witness.” Participants commented frequently that these groups were the first time they had engaged with a community with lived experience, and this shared experience was frequently highlighted in the responses from individuals as one of the most unique and important aspects of SCI Thrive. As such, we have discussed the identified themes as both unique themes as well as highlighted how they overlapped with Bearing Witness (see Table 3).

**Table 3:** Themes identified through forum content.

Theme	Straight forward	With bearing witness
Resource sharing	“I also want to add a couple resources for those folks who are interested in starting a meditation and mindfulness practice. I highly recommend a CD guided meditation called…it fits in well with our setting goals homework. There are lots of guided meditation CDS/videos on you tube as well. I use subliminal CDs in my nighttime routine. I simply play a positive, healthy message subliminal CD while I fall asleep…”	“I’m a C3 incomplete who seemed to dodge a bullet as I’ve been able to regain the ability to walk, or at least some semblance of. One thing that really helped me was the NuStep machine. I’m not sure if you have access to one…but if you do, I strongly encourage you to set up a routine. In 6 months…I went from 500 ft to 3,000+ft using my walker… Yes, I still use, and need, frequently. It's super easy to transfer from chair to machine since the seat swivels….Most big league gyms…have too.”
	“I run a support group for women who use wheelchairs. If you are interested in joining us, send an email…let me know what your disability is. Women only.”	“My long term goal was to exercise. In the last assignment I used the words, “exercise more” and what I meant was to exercise period because I don’t exercise at all, although I make sure I move around enough. My short term goal was to start out slow basically and I was able to do that Friday-Monday, which is more than I thought I’d do. I found 2 great wheelchair exercise videos that make me feel great after. I’ve done them on and off but I kind of created a routine the last 4 days so that I could reach my goals and stop making excuses or procrastinating. I am guilty of both unfortunately. These are the videos I found extremely helpful….”
	“Hi everyone, A positive of COVID-19 is that all the conferences are online! If you are interested in accessibility and inclusive design, there's a virtual conference you can check out … Their talks are streamed on YouTube…”	“Hi everyone, Hope you are well. Since some of us have shared that we have exercise as one of our goals so I want to let you all know that [DEL] is holding a virtual exercise/yoga sessions and it is open to everyone. If you are interested please check them out here…Hope you will find this helpful. Stay safe and healthy.”
Skill Building (specific SCI Thrive skill in bold)	“Thankful to study this section today and yesterday, Perfect timing. Helpful in the meeting today. Walked in a few minutes before the meeting started. Signed in as a local commentor [sic] and was the first speaker to be called up. Regarding new parking areas in our little town… Used **“I” statements**. Started by thanking them for what they did on the corners, making easy access to siderales [sic]. Then, spoke about the limited parking that is now available which makes me have to walk a lot further, while not a good walker. Plus, the curbs are very high to climb. I felt that the verbiage used was assertive and grateful that this class showed up today.”	“I've always seen myself as a positive person, but there are things about SCI that just sometimes suck. occasionally people will tell me to “think positive” and I find that irritating, because even though I am positive person sometimes it is just overwhelming and all consuming and negative situations we find ourselves in. I like the idea of **alternative thoughts**, to me it seems more realistic than "be positive".. Its [sic] realistic, but also thoughtful thoughts.”
	“this was an interesting exercise for me. My tension was low at the beginning, and even lower at the end. The persons [sic] voice alone on the recording is relaxing. Focusing on a part of the body and **breathing** as instructed did provide me with a relaxed state.”	“it is not always easy to accomplish **goals**… So don't beat yourself up too much sounds like you did accomplish a lot even with your UTI. The sunshine is very healing and will help you with those challenges as well. Take care and be safe”
	“**Autogenic Relaxation**: This was not my favorite technique. I found it hard to actually feel relaxed after thinking about being tense and heavy with the steps in this exercise. It was worth a try for something new but I don't anticipate doing this exercise much on my own”	“you are not alone, i used to be very outgoing, now i just want to stay home with my family but i'm tyring [sic], my **goal** is to find new things that give me a sense of independence and fullfillment [sic] thank you for sharing”
Problem solving	“Thank you for sharing, I have decided to implement your tactic of prioritizing exactly what assistance you will need … rather than asking for assistance to get it done faster. This has been great for me so far, and I'm still working on it”	“Wow I love such sense of understanding that I get from this group. Thank you, I will definitely keep those suggestions in mind and I have been in the process of exploring what exactly is out there in order for me to change my environment. I am a person with much value in silence and privacy, so I feel you can only imagine what I'm going through internally right now. Thank you so much for the suggestions”
	“I would explore ways to move out of your parents home, perhaps a group home where you have supporting staff. This may take months or longer. I think you will find your parents are relieved not to worry about your care constantly.”	“This [is] helpful to me in understanding my spinal cord injury. Initially prior to this course I thought I could achieve a complete recovery. I have now learned that it is unlikely. I have been enlightened by the group members. Hearing about their spinal cord injury, and for how many years they have been living with it. They are wonderful people living their lives. Therefore, I come away inspired”
	“I always ask in-home care providers when I hire them to give me a month notice if they plan to leave the position. I recently had an employee provide me with only 2 weeks notice. This is very frustrating for me and made me anxious in locating a new person in that short of time period. I decided to draw up an informal contract that outlined the job description, requirements for time off and time necessary for ceasing employment. I understand is not legally binding nor do I wanted to be yet I would like my in-home caregivers to be professional enough to take the position seriously.”	“Wow! What a heart felt description of being in your situation. You really are amazing! You break my heart with the thought you are panicking. I once had a horrible panic attack and I can move better than you yet it paralyzed me even more. First, know u are definitely not alone. We are all in this together and we gotta be there for one another as [another group member] eloquently wrote. I support and understand you in your struggle. Pisses me off that we have to deal with our conditions and then have to deal with so many other problems besides the physical disability. But remember when those thoughts start to invade your brain, say to yourself "I got this." You have dealt with this challenge for awhile now and you will continue to handle it. It will get better. A PCA will become available, things WILL work out. Take it one moment at a time because if you try to look at the whole picture it seems overwhelming.”

### Resource sharing

One of the most tangible and basic uses of the forum was a setting for individuals to share SCI specific resources that had been helpful for them. Often resources were related to skills discussed in the SCI Thrive course but also expanded beyond these specific course topics, including exercise/adaptive sports, mindfulness, and caregiving. In many cases, posts focused on accessing a specific, established resource. These types of posts were valuable, and many but not all were specific to an individual with lived experience of SCI. Many participants commented that this was the first time that they were able to learn from and share with others with an SCI. This is also a setting in which individuals had the opportunity to receive information from peers rather than from professionals. Many posts reflected this shared experience by including validation of the SCI experience and expressions of care such as, “stay safe and healthy.” These types of posts included the element of Bearing Witness. In this category, Bearing Witness also appeared in the form of storytelling especially illustrating how making and meeting one's goals led to improvements in health.

### Skill building

SCI Thrive was structured to guide participants through the process of creating goals and following-up on their progress by practicing a series of related skills (see Table 1). Goals were both connected explicitly to how to manage life with SCI and addressed quality of life more broadly. A common theme was that the accountability associated with SCI Thrive encouraged group members to complete the skill building exercises more frequently than they would have otherwise.

Participants were encouraged to discuss their experiences with each exercise in the online forums. The mindfulness practices–guided imagery, breathing, and autogenic relaxation–were particularly tangible skills to comment on. People entered the program with varying levels of experience with breathwork and meditation and commonly shared other examples, especially of breathwork, that they had practiced. These and other skills were discussed as tools to manage SCI-related issues that cause anxiety and stress including hiring and managing caregivers, traveling, and pain-management. There was explicit acknowledgement of the connection between physical and mental health with frequent mention of common SCI health complications that impact quality of life including nerve pain and bowel and bladder management. One participant described the use of the mindfulness skills as “mindful medication.”

Individuals used the skills to engage in mean making and articulating a robust narrative of self, including and beyond disability. Here there was significant presence of bearing witness. There was both general acceptance of the physical body and life circumstances post-SCI and a coming to terms with the changes to one's situation. Importantly, the life narrative was not exclusively defined by SCI, but the forum conversations hinged on changes in how one approaches the world due to SCI-related differences (i.e., lack of independence, and the need for more planning and self-advocacy). Participants expressed empathy (“there are things about SCI that just sometimes suck;” “you are not alone”) and encouragement (“don't beat yourself up too much”) in relation to the life challenges that accompany SCI.

### Problem solving

In the forums, the cohort worked together to brainstorm or problem solve everyday problems associated with SCI such as managing one's care and communicating with providers. This took the form of practical, peer-focused problem solving that involved sharing a challenging situation with the group while members responded by simply helping think through an issue. Participants shared strategies they used in similar circumstances and best practices advice. As with resource sharing, there was acknowledgement of the need for different resources or strategies due to the conditions surrounding SCI.

Often this co-occurred with a tone of empathy that distinguished it beyond just problem solving, adding the component of Bearing Witness. Expressions of care (“We are all in this together and we gotta be there for one another”) and admiration (“You really are amazing!”) were apparent. Participants expressed an emotional connection through the process of learning about and responding to the problems others faced. This provided validation for their own struggles and reflected the psychological experience that the participants were sharing. In describing the benefits of this empathic problem solving, participants used terms such as “greater understanding,” “enlightened,” and “inspired.”

### Bearing witness

As highlighted in the previous discussion, Bearing Witness was a pervasive theme that was integrated into other identified themes. In addition to being a component of Resource Sharing, Skill Building, and Problem Solving, as highlighted in Table 1, Bearing Witness was also a standalone theme of interaction that often occurred outside of the structured content of the SCI Thrive course. Within this spontaneous and rich theme, we identified three sub-themes: Connectedness, Words of Encouragement, and Shared Problems (see Table 4).

**Table 4:** Themes within bearing witness.

Theme	Participant quote
Connectedness	“I like that we will be able to all get to know each other. I've only met one other person with sci, although I've also had some contact with people in forums, that's felt rather impersonal”
	“Personally, I keep coming back to your story because it gives me hope, and reminds me what strength and preservation can offer. Being new to the world of limitations i currently feel (and perseverate on) from my SCI there is also boundless hope in your words.”
	“going through this course and reading about the issues others have to deal with (regular bowel care program, etc.) in addition to the emotional and pain and relationship issues that sci can bring on, I am deeply moved. Some things in my body seem to be getting worse, and thinking of how others here persevere gives me resolve that I too can deal with what the future brings.”
Words of encouragement	“I am so impressed with your vision. Doing your best to take responsibility for your care helps you as much as it helps your wife, as I'm sure you know. Also, continuing to pursue your art is wonderful on many levels. You are giving to the world—corny of me, but true”
	“Good for you for researching your options and continuing to look forward to a better living situation. You're facing a lot right now, it sounds like there isn't an aspect of your life that gives you peace and lets you recharge, and the people around you aren't the ones you'd choose if you had the luxury of choosing. THAT is stress! I feel for you and am impressed that you have the strong will and determination that you have. It will serve you well, and I hope your situation improves soon”
	“I think it is very healthy to acknowledge your grief and try to deal with it. I know it can be overwhelming at times but it sounds like it is a healthy process for you.”
Shared problems	“I too deal with a boatload of memory issues. I was on gabapentin for 13 yrs. Not to mention I had a TBI and several concussions. So, I guess what I'm saying is I hear ya. It sucks!”
	“I MADE IT FOR MY (I TELL MYSELF, ITS [sic] AN IMPORTANT APPOINTMENT!) ONLINE bariatric HEALTH SUPPORT GROUP FOR THE FIST [sic] TIME THIS MORNING AND I AM PREPARING FOR A STRESSFUL HEARING WITH SSI IN THE MORNING. SO JUST HAVING SOMEONE TO TELL MAKES me feel like I'm doing good today!”
	“Being honest and assertive about my changing needs and how I am actually feeling when being minimized by my condition, is a very valid, but difficult path. Nobody but other SCI people really understand the struggles, and it can be exhausting to always be so honest and communicative”

As with other themes, many individuals highlighted that this was their first opportunity to engage with a peer group. Within the sub-theme of Connectedness, participants shared that hearing from others about their SCI experiences conjured strong emotions of hope and resolve. This connectedness and shared experience appeared to be a more powerful source of validation than can be offered by a provider without lived experience. If the Connectedness subtheme highlighted the benefit of the shared experience for the individual, then the sub-theme of Words of Encouragement appeared to be an expression of how individuals reached out to others, both highlighting how story telling has inspired them but also noting successes and encouraging perseverance. And both Connectedness and Words of Encouragement were often linked to Shared Problems. While this was similar to Problem Solving, it was distinct in that the emphasis of these discussions were not on how to resolve an issue, but rather validating the unique challenges experienced while managing SCI and acknowledging the rawness and intensity of the emotions that can accompany these challenges.

## Discussion

This study aimed to understand components of peer interactions between individuals with SCI engaged in an online self-management program. There was evidence of expected themes related to the course content, such as Resource Sharing, Skill Building, and Problem Solving. Beyond just the practical engagement with course content and each other, a theme of rich emotional connectedness and support was identified, which we called Bearing Witness. Bearing Witness included a collective response to course work, including validation, normalization, and sometimes feedback. This theme permeated all themes but also occurred in the absence of practical discussions.

Themes identified in the current study are similar to findings of qualitative studies examining peer interactions for a wide range of chronic conditions. A scoping review of peer support interventions across a wide range of chronic conditions found that emotional support, similar to bearing witness, was particularly valued by mentees (13). Similarly, an evaluation of unmoderated social media interactions for individuals with mental health symptoms also found themes of problem solving, resource sharing, and emotional support (14). Specific to self-management or health education interventions, individuals participating in dual-diagnosis substance use groups reported greater knowledge acquisition from peer-led group compared to professional led (15). Participants reported similar themes of emotional support, feelings of empowerment, and freedom to speak that stemmed from the shared experience of the peer group. Similarly, a systemic review of peer support within self-management interventions following stroke also found that participants especially valued the shared experience and productive social comparison (16).

The current findings paired with the broader literature on peer support suggest that the benefit of peer groups is not just about specific knowledge within a peer group. It is also about caring for and supporting each other in a very specific way that isn't possible without the peer connectedness. Common ground allows for genuine validation and empathy. Similar themes of connection and Bearing Witness may be present in other resources, such as support groups. However, support groups are usually primarily focused on Bearing Witness and rarely pair the emphasis on behavior change along with the validation from the perspective of lived experience. Within the context of behavior change, this validation is particularly important. Motivational interviewing, a widely implemented intervention for health behavior change, emphasizes the importance of collaboration and validation before engaging in problem-solving or giving advice (17). In this peer community, the initial validation happened organically and was frequently cited by participants as being the most important factor and benefit of the intervention. Given how powerful individuals reported this validation being, it is possible that subsequent interventions to promote the behavior change may be more powerful with this kind of validation first.

In addition to potentially serving to amplify the behavior change intervention, the shared lived experience may have allowed participants to connect with each other more quickly. Rather than needing to give basic information about SCI that would be required for connection with individuals without SCI, talking with peers about SCI related experiences meant that all involved already understood much of the logistical or baseline information that allowed them to move toward empathy and connection more quickly. Perhaps the Bearing Witness component may be the most important component of peer interaction and allowed participants to coalesce into a community. This is particularly relevant given the timing of this intervention, which occurred throughout the early COVID-19 pandemic. While the broad population experienced increased loneliness during this time period (18), findings from our trial suggest that the participants in SCI Thrive did not experience a significant decrease in social integration (8). Perhaps this stability could be related to the experience of shared peer community.

The current study is limited by several factors. First and foremost, the current study was focused on identification of themes within peer interactions. As such, qualitative analyses and themes identified have not been linked with quantitative outcomes. While both the research team and peer leadership team propose hypotheses about the benefit of these interactions, future research needs to more fully examine the impact of these themes. Regarding the content analyzed, thematic analysis only included the participant forum posts and excluded post by peer leaders. As such, we are unable to determine the origin of discussions. While many discussions may have started in the online forum, some of the discussion are certainly a continuation of interactions during the video conferences. Finally, the current study is limited by the demographics of the individuals who participated. The study population was primarily white, older, and relatively educated. A larger portion of the population was from the western region of the country, and many individuals living in metropolitan areas. As such, the current themes may not be reflective of a more diverse patient population. Furthermore, participation required individuals to have computer or smartphone access and literacy. Individuals with lower literacy may have been less able to participate, and therefore their experiences are not reflected in the current qualitative analyses. As individuals with less technological access or ability may be more likely to experience other disadvantages, this is an important population to include in future interventions and research.

Even with these limitations, the findings suggest that interventions can benefit by harnessing the power of the peer group. The content and structure of the intervention brought individuals to the group, but anecdotal evidence and themes identified through this study suggest that the culture and shared experiences and supportiveness was equally important as the factual or skills-based information provided. Future research on SCI self-management should continue to explore integration of peer community when possible. Connectedness and shared experience may increase participant receptivity to educational material and amplify motivation for behavioral change. While the current study focuses on SCI, similar effects could be present for other groups with chronic medical diagnoses and conditions.

## Data Availability

The datasets presented in this article are not readily available because data consists of narrative information provided by research participants. As there may be potential identifiers within the dataset, it cannot be made publicly available. Requests to access the datasets should be directed to jeanneh@uw.edu.
